# Professionals’ perceptions and current practices of integrated palliative care in chronic heart failure and chronic obstructive pulmonary disease: a qualitative study in Belgium

**DOI:** 10.1186/s12904-018-0356-7

**Published:** 2018-08-24

**Authors:** N. Siouta, P. Clement, B. Aertgeerts, K. Van Beek, J. Menten

**Affiliations:** 1 Department of Experimental Radiotherapy and Palliative Care, UZ Leuven, Campus Gasthuisberg, Herestraat 49, 3000 Leuven, Belgium; 2 Department of Experimental Oncology, UZ Leuven, Campus Gasthuisberg, Herestraat 49, 3000 Leuven, Belgium; 3 Department of Public Health and Primary Care, Academic Center for General Practice, Kapucijnenvoer 33, 3000 Leuven, Belgium

**Keywords:** Heart failure, Pulmonary disease, Chronic obstructive, Palliative care, Quality of health care

## Abstract

**Background:**

Patients with Chronic Heart Failure (CHF) and patients with Chronic Obstructive Pulmonary Disease (COPD) share similar symptom burden with cancer patients, however, they are unlikely to receive palliative care (PC) services. This article examines the perceptions of health care professionals and the current practices of integrated palliative care (IPC) in Belgium.

**Methods:**

Cardiologists and pulmonologists, working in primary care hospitals in Belgium, participated in this study with semi-structured interviews based on IPC indicators. One researcher collected, transcribed verbatim the interviews and carried out their thematic analysis. To increase the reliability of the coding, a second researcher coded a random 30% of the interviews.

**Results:**

A total of 22 CHF/COPD specialists participated in the study. The results show that IPC and its potential benefits are viewed positively. A number of IPC components like the holistic approach (physical, psychological, social, spiritual aspects) via multidisciplinary teams, prognosis discussion and illness limitations, patient goals assessment, continuous goal adjustment, reduction of suffering and advanced care planning are partially implemented in several health centers. However, PC specialists are absent from such implementations and PC is still an end-of-life care.

**Conclusions:**

Misconceptions about PC and its association to death and end-of-life appear to be decisive factors for the exclusion of PC specialists and the late initiation of PC itself. The implementation of IPC components is not associated to PC, and as such, leads to suboptimal results. Improved education and enhanced communication is expected to alleviate existing challenges and thus improve the quality of life for the patients.

## Background

Chronic Heart Failure (CHF) and Chronic Obstructive Pulmonary Disease (COPD) are two prominent life-threatening chronic diseases with a worldwide prevalence of 23 and 3 million respectively [1, 2]. Patients with CHF/COPD face heavy physical and psychosocial burdens, comparable to cancer patients [3–6]. Although treatment for CHF/COPD is not curative but focuses on symptom management and life prolongation, survival rates have increased with time [7]. The Palliative Care (PC) needs of these patients are subtle and require a more integrated, systematic and sustained approach to the provision of high-quality care [7].

Integrated Palliative Care (IPC) involves bringing together administrative, organizational, clinical and service aspects in order to realize continuity of care between all actors involved in the care network of patients receiving palliative care.^1^ It aims to achieve quality of life and a well-supported dying process for the patient and the family in collaboration with all the care givers [8]. IPC targets patients with both malignant and non-malignant disease [8, 9] and empirical studies have demonstrated that it improves the quality of life of CHF/COPD patients and their families by reducing symptom burden and the frequency of hospitalizations and by addressing their goals and needs throughout the disease trajectory [10, 11].

There is conclusive evidence that patients with CHF/COPD are much less likely to receive PC in general, than cancer patients [12, 13]. In fact, only less than 20% of CHF/COPD patients ever have access to PC services whereas for cancer patients the corresponding percentage is usually higher than 50% [14–16]. Consequently, the percentage of CHF/COPD patients who will benefit from IPC either early or later in the disease trajectory is markedly less [8].

The inequity in the provision of PC between patients with CHF/COPD and cancer has attracted considerable attention. Several studies have tried to identify the roots of this inequity by examining the perceptions of the different medical specialists on PC. These studies have revealed the existence of several restricting factors. Among these factors, reported in the literature, are the limited PC knowledge of healthcare providers [17–20], misperception of PC as an end-of-life care [20], complexity of prognostication, especially of non-malignant diseases, and difficulties with the timing of referral [19–24], perception of CHF and COPD as “manageable” chronic diseases [22, 25, 26] and inadequate communication and collaboration between the involved medical disciplines [8, 27–29].

On the other hand, much less is known about perceptions of medical specialists on IPC for patients with CHF/COPD. Moreover, current practices remain poorly understood. However, as IPC represents a change of mindset in the provision of PC and authorities are progressively advocating in favor of its incorporation, it is timely to examine both perceptions and current practices especially prior to the development/implementation of specific guidelines. As perceptions of specialists can be affected by a country’s culture, and given that even within the European Union there is no uniformity on IPC current practices, it pertains to investigate these topics locally in the level of countries.

The present study aims to perform a step in this direction by examining perceptions of cardiologists and pulmonologists and current practices of IPC in patients with CHF/COPD in Belgium.

## Methods

### Design

In Belgium, acute wards of cardiology and pulmonology develop their own PC strategies. These strategies are shaped by and reflect the perceptions of specialists (cardiologists/pulmonologists). Although its actor in these wards has his/her own perceptions on IPC, a natural first step would be to focus on the specialists and in particular on those cardiologists and pulmonologists who specialize in treating CHF and COPD patients respectively. In order to describe their perceptions on integration of PC in patients with CHF and COPD, a qualitative descriptive study was deemed more appropriate. The individuals that agreed to participate in the study provided their informed consent via e-mail and their interviews took place in person or via phone. The reason for studying CHF/COPD in a combined fashion is the fact that these two non-malignant and high prevalence diseases share heavy physical and psychosocial symptom burden, similarly complicated disease trajectories and they are not subject to curative treatment [3–6]. Consequently, one expects that CHF/COPD specialists will share similar views on the role of IPC for these diseases.

### Inclusion criteria

Eligible participants were cardiologists, specializing in CHF and pulmonologists, specializing in COPD (treating CHF and COPD patients respectively in the wards). These participants had to be employed in public hospitals in Belgium (both outpatient and inpatient settings, including academic medical centers and different community based hospitals). Further, they had to be able to be interviewed in English.

### Sampling and setting

For this study we initially aimed for a total population purposeful sampling [30]. For the identification of the eligible population we used a two-fold strategy. First, we contacted (via email and phone) the directories of every public hospital and we inquired their cardiologists and pulmonologists that were specializing in CHF and COPD. Second, when contacted these eligible specialists and besides asking for their participation to the study we also asked them for possible chain-referrals [31]. All eligible participants were contacted via email and/or phone, followed up by a second reminder a month after the initial invitation. Our contact efforts stopped when we reached data saturation. The inclusion period of the study lasted for November 2016 until April 2017.

### Data collection

The main researcher (NS) collected the interviews with an electronic audio recorder in person (in the hospital setting) or via phone. Based on previous work a semi-structured interview containing 10 questions was developed [8, 32]. These questions are linked to the IPC indicators used in previous studies in order to measure the content of integrated PC in guidelines and pathways of cancer and CHF/COPD in Europe [8, 33]. The questions are shown in Table 1 in succinct form. In practice (NS) used these questions as starting point of discussion to encourage the participants to reflect and elaborate on the topics. The participants were not given the list of questions.

**Table 1:** Core questions for the semi-structured interviews

1	Are patients usually informed about the prognosis of their disease? How easy/difficult is that?
2	Is it needed to have holistic assessment and who should do that? When should the holistic assessments be applied?
3	When should palliative care be initiated? For what is the patient referred?
4	Is it necessary that patients’ goals are explored?
5	Do goals change during the disease trajectory?
6	What are the most frequently used interventions for suffering reduction?
7	Is advance care planning useful for cardiology/pulmonology? In what percentages of patients it is used?
8	When is the palliative care team involved?
9	Are there any specific recommendations on what to do in the patient’s last hours of living?
10	Are there any specific recommendations for support of the family of the patient post-mortem? Is grief and bereavement care present?

### Data analysis

The data collection and analysis was cumulatively iterative. The interviews were transcribed verbatim and were independently analyzed and coded with the NVivo 11 software program by the main researcher (NS). The interview data were analyzed based on thematic analysis [33]. One researcher (NS) read all the transcripts, coded sections of text and set up an initial list of codes that lead to a broader range of identified codes. To increase the reliability of the coding, a second researcher (KVB) coded a random 30% of the interviews. The two researchers met regularly in order to review the coding structure, to ensure codes were applied in a consistent manner and to resolve any possible discrepancy. Anonymity of the participants was preserved throughout the analyzing and coding process [34]. Additionally, three authors (NS, KVB, JM) discussed extensively the evolving themes of three randomly selected interviews to ensure further validity of the procedure. Data saturation was reached independently for both CHF and COPD specialists following the theory that data collection stops when additional interviews do not add more to the results of the prior findings [34]. Results are presented and discussed in a combined fashion when appropriate.

## Results

We invited electronically via e-mail 312 cardiologists (CHF experts) and pulmonologists (COPD experts). The 22 individuals who participated in the study provided an informed consent via e-mail and the interviews took place in person (16/22) or by phone calls (6/22). Three (3/22) were from the region of Brussels (French/Dutch speaking), eighteen (18/22) from Flanders (Dutch speaking) and one (1/22) from Wallonia (French speaking).

The characteristics of the participants are presented in Table 2. The mean duration of the interviews was 32 min (range 20 to 50 min). Our analysis followed the scheme of the themes of the IPC indicators provided in Table 1.

**Table 2:** Characteristics of study participants

Characteristics	Full Sample	Cardiologists	Pulmonologists
N	22	12	10
Age			
50–69 years	8	5	3
30–49 years	14	7	7
Gender			
Female	6	4	2
Male	16	8	8
Practice setting			
Academic	9	5	4
Non academic	10	4	6
Both	3	3	–

### Are patients informed about their prognosis? How easy/difficult is that?

The majority of the participants agree that it is important to discuss the prognosis with the patients and they do this in practice. The core reason for doing so is the belief that it is honest and equally important to inform both patients and family on illness trajectories and treatment possibilities and limitations so that they can adjust their expectations.

The analysis revealed a large variation concerning the timing when prognosis discussions should take place. Some suggested that this discussion is a progressive process that cannot take place in the first contact but should gradually start in the subsequent meetings when the physician is more aware of the psychological reaction of the patient. Few participants however declared that discussions on prognosis should take place while communicating the diagnosis of the disease or immediately after every re-hospitalization or after an exacerbation of the disease. Few participants suggested that these prognosis discussions should occur later in the disease trajectory e.g. when a patient has a GOLD stage D diagnosis, because it is perceived as very confronting and terrifying for the patient.

*“You have to discuss it with the patient, but you cannot do it in one encounter. You have to feel how she/he is coping with that issue and try to go for a personalized choice together with the patient”. Cardiologist 7*

The majority of the participants found it difficult to discuss about prognosis because of the complicated and unpredictable nature of the disease (both CHF and COPD) while it is easier to predict the prognosis for oncologic patients.

*“Now it’s not easy to say….it’s not easy to determine who’s going to die in cardiology. It’s not like in lung cancer where we have scientific evidence that survival is mostly between 4-6 months”. Cardiologist 3*

*“For lung cancer patients when you see that the treatment doesn’t work and that the patient gets more and more tumors then you know you call in the PC, but in COPD the interventions are not clear e.g. you one cannot check it on a CT scan or so…”. Pulmonologist 3*

Another factor to render such discussions as challenging is that PC is perceived as a “bad word”. As it turns out, most patients and healthcare providers have connected this word with “death” and “dying” and also with the care that is mainly provided to cancer patients, but not to CHF/COPD patients.

*“And to some certain extent I prefer the word “supportive” than “palliative”. If I use the word palliative to a patient then they will think “oh, I’m immediately dying” and then it’s a problem”. Pulmonologist 6*

### Is it needed to have holistic assessment and who should do that?

All participants agreed that the provision of a holistic approach is crucial for the care of patients with CHF and COPD.

*“Yes, of course. The medical treatment alone is not a holistic approach” Cardiologist 6*

*“Yes, I find a holistic approach very useful and that a patient can highly benefit from it”. Pulmonologist 5*

Almost all interviewed participants reported that the holistic approach for the hospitalized patients is provided by a multidisciplinary team (MDT). This team consists usually of medical specialists, nurses and specialist nurses, a social service expert, a psychologist, a physiotherapist/kinesiotherapist and a nutritionist. The MDT’s meet on a weekly basis. Only few participants mentioned the presence of PC experts at the weekly meetings. In most meetings, the psychologist is not a standard member of the MDTs, but is available on demand.

### When should palliative care be initiated?

Participants gave a variety of answers. Most mentioned that PC should start when the curative treatment is no longer realistic. Some reported that PC should be initiated if many repeated hospitalizations occur within a short period of time or when there is currently no longer an acceptable quality of life for the patient. Most participants are aware that PC is implemented frequently at the end of life but that it should be ideally initiated earlier in the disease trajectory for the benefit of the patients.

*“Normally, this happens pretty late, mostly at the end of life of the patient. I think that it should be initiated even earlier, let’s say even from the moment that a patient is diagnosed with COPD so they can benefit as much as possible, because PC is not only for the end-of-life”. Pulmonologist 5*

One of the reported difficulties for defining the timing of PC initiation is the unpredictability of these diseases and the definition of PC itself. Some of the participants expressed that they had difficulties understanding what PC exactly is.

*“I also don’t know what do we understand as PC. Is it the life ending? Is it the active life ending? Is it a passive life ending?”. Pulmonologist 8*

### Is it necessary that patients’ goals are explored?

There is a unanimous agreement on the necessity to explore the goals, wishes and desires of the patients. All participants find it very important to explore and discuss patient’s desires and wishes whenever possible, because the treatment goals should be adjusted accordingly and be adapted based on the stage of the disease. The lack of time is also mentioned as a potential barrier for not assessing the goals more regularly in reality; this is more apparent in the larger hospitals were physicians seem to be overloaded, work in teams with frequently need for replacements of each other and modus operandi does not allow for extensive interpersonal communications with the patients.

*“Yes, that’s important. A treatment of a patient is not a one-way communication, it’s a two-way communication”. Cardiologist 7*

*“In the local hospitals care is more personalized…”. Pulmonologist 9*

### Do goals change during the disease trajectory?

All participants suggest that the goals and wishes of the patients do change during the disease trajectory. Most mention that both physicians and patients change and adapt their expectations according to the changes in the disease trajectory. The patients appear to have fewer expectations while the disease is progressing. Some participants commented that the duration of the patient’s/specialist’s relationship is important; the longer you know the patient the better you are informed for and keep up with their goals and wishes.

*“Yes, we see it changing as the disease progresses. They have less expectations as the disease progresses”. Cardiologist 9*

### What are the most frequently used interventions to alleviate suffering?

Almost all the participants suggest that morphine and sedatives are used to reduce physical suffering in advanced stages of CHF and COPD. Other frequently used interventions are the administration of diuretics (for CHF), or oxygen (for COPD). For the relief of psychological suffering, some participants mention the use of anxiolytics or benzodiazepines along the provision of psychological or spiritual support if this is asked by the patient. Some participants contact the PC team in case they feel that they comfort the patient insufficiently.

*“We provide morphine and if we need to provide sedation we contact the geriatricians and the PC Support team to come and give some instructions to our team. But my opinion is that we always give sufficient diuretics.”. Cardiologist 11*

*“We supply oxygen for breathlessness and if oxygen doesn’t help we give morphine, because that reduces the pain and the dyspnea. And sometimes also anxiolytics like Xanax to reduce stress”. Pulmonologist 2*

### Is advance care planning useful for cardiology/pulmonology? In what percentage of patients is it used?

Almost all participants suggest that advance care planning (ACP) is central/mandatory to CHF/COPD patients. Further, the majority of the participants reports that ACP is realized in practice. However most of the participants could not provide the percentage of patients that receive ACP. There is no consensus on the timing of ACP; some participants mentioned that ACP is applied early in the disease trajectory while others initiate ACP close to the end-of-life.

### When is the palliative care team involved?

Most of the participants report that in reality the PC team is not involved in the treatment of a patient with CHF/COPD or is involved much too late in the terminal phase of life. Some participants involve the PC team when the curative treatment is no longer effective or when the patient had multiple admissions in a short time. Few highlight the importance of involving PC earlier in the PC trajectory and not only when they are invited because the patients ask for it or because there is no longer a treatment option.

*“I think PC is a bit underrated in CHF, because we tend to try to cure the patient till the last time. And also you need to have some guts to be able to talk about it with the patients, cause they are not always aware of it”. Cardiologist 10*

*“We should ask the involvement of PC for these patients, but we obviously don’t do it so much, but we call them in for the last months”. Pulmonologist 7*

### Are there any specific recommendations on what to do in the patient’s last hours of living?

The majority of the participants do not use any specific protocol or guideline in the last hours of a patient’s life, but they do what they assess as necessary to be done. The emphasis on a personalized care is highlighted especially in smaller hospitals. Some participants use specific checklists and protocols that they follow at the end-of-life of a patient. Others call the PC team for assistance how to proceed further. Few participants think it is useful to consult colleagues for help in making decisions for the last hours. Many participants would welcome a protocol or a guideline on how to deal with the last hours of life of patient.

*“I don’t think we follow specific guidelines or a specific protocol. We do what it feels best to do. In the local hospitals care is more personalized and not so standardized”. Cardiologist 11*

*“We have a specific scheme that we try to follow, of course it may differ a bit from one doctor to the other”. Pulmonologist 9*

### Are there any specific recommendations for the bereavement support of the patient’s family?

The results revealed that most of the bereavement support is provided by the specialist physician and nurses who cared for the patient with listening, giving information and answers to questions. For this reason, some participants find it mandatory to discuss the expected dying process with the families before the patient dies. It is usually uncommon for the families to seek psychological support post-mortem. In case they do look for support, few participants mention the availability of a psychologist and in fewer cases the provision of spiritual care. Another aspect that inhibits the provision of grief and bereavement support for the specialists is the variety of religious and cultural backgrounds.

*“Yes, so we first we give them their time. Then the doctor that takes care of the patient will go there and then the nurse that takes care of the patient will talk to the family and ask what they need and what they want. If they need to talk to a psychologist, then we will contact the psychologist of the ward and the social service”. Cardiologist 12*

*“As physicians, we need to be there at the end of life too, it’s important to provide personalized care”. Pulmonologist 7*

## Discussion

This is the first study that documents perceptions of cardiologists and pulmonologists and current practices of IPC in patients with CHF/COPD in Belgium. The results show a favorable view towards IPC and its potential benefits. Indeed, the holistic approach via MDT’s, discussion of prognosis and limitations resulting from the illness, assessment of patient’s goals, continuous goal adjustment, reduction of suffering and ACP constitute components of IPC have already been implemented (even partially) in many Belgian health centers. The occurrence of discussions on prognosis, in particular, is very interesting since previous studies have reported that patients with CHF/COPD are quite unlikely to get engaged in such conversations [35–37].

However, even though, some of the IPC’s components have already been (partially) implemented, PC specialists are seldom involved and PC, in particular, is not initiated until the end-of-life. In other words, we have the conundrum that PC aspects are integrated alongside standard treatment without the involvement of a PC team. Second, even though participants explicitly stated their preference towards an earlier initiation of PC, they do not apply this in practice. In turn, this implies that PC practices are quite confined and cannot grow easily to their full potential, not least because the specialized knowledge of PC specialists is utilized. Accordingly, this may impact the quality of life of patients and their families since the application of these IPC components might be suboptimal. For example, ACP in the end-of-life stage does not offer substantial benefits.

Based on the collected perceptions, we find two reasons for the explanation of these conundrums.

### Misconceptions about PC and its role

When prompted to talk about PC, participants use cancer as a reference and comparison point. In other words, the fact that PC was initially applied in cancer continues to affect the mentality of specialists outside oncology. Further, even though the World Health Organization (WHO) promotes a more integrated approach to PC, the participants perceive PC as an end-of-life care. Consequently, the fact that PC is limited to the alleviation of suffering and symptom control during end-of-life and not concerned with other aspects of quality of life appears as a justifiable situation.

These findings are typically reported in studies focusing on PC for patients with non-malignant disease [20, 38]. Collectively, they suggest that there is i) a lack of communication concerning the overall role and benefits of PC in the quality of life of patients and ii) insufficient training and education of specialists in the fundamentals of PC.

### PC has a “bad name” reputation

The participants mentioned that the word “palliative” is a bad word that results in undesirable confrontation with both patients and their families. Given that prognosis discussions are already challenging in CHF/COPD and that the general public image of CHF/COPD does not associate these diseases with death, participants are reluctant to mention the existence of such services, even more so early in the disease trajectory.

This finding is not surprising. Even in oncological wards, where PC is more standardized, the term “palliative” is associated with death and end-of-life and has been empirically demonstrated to adversely affect inclusion rates and early referrals [39]. In fact, empirical studies have found that renaming PC to “Supportive Care” can have a positive impact on both medical staff and the patients and leads to improved inclusion and referral rates. [40–42]. Berry et al. go far enough to suggest rebranding PC so that this “shadow” is removed [43]. It is not clear whether a change in the terminology will have a positive impact in CHF/COPD patients since Randomized Controlled Trials (RCTs) have thus far been restricted to oncology. Moreover, PC is inherently related to difficult concepts (e.g. prognosis discussions, ACP decisions) and this is independent of the name of such services. In other words, even if PC was renamed one would still need to address these difficult issues.

It is important to comment on bereavement care and support for the families post mortem. The analysis clearly shows that there is a lack of bereavement support. Conversations that aim to resolve questions with family members are not available and psychologists and spiritual caregivers are very rarely available. The low level of bereavement support is a recurrent finding that can even be traced in guidelines/pathways for PC in CHF/COPD. The systematic review by Siouta et al. shows that recommendations on bereavement support was among the weakest issues in the existing guidelines/pathways [8]. Consequently, bereavement support appears to be an important component that has not yet the necessary attention.

#### Limitations

The first limitation of this study concerns our sample. We have opted for a total population purposeful sampling, but only a fraction of the population agreed to participate. We are not aware of the reasons why eligible participants refused to participate since they ignored our invitation at the first place. Consequently, we can only speculate on the reasons of non-participation. It is possible that eligible medical specialists that did not/did participate in the study chose to do so because they were unfamiliar/familiar with IPC. If this is the case, then the generality and generalizability of our results is affected. In fact, even though we reached data saturation, sampling biases would be present if eligible specialists based their decision to participate on their knowledge of IPC.

A second limitation is that the interviews were conducted in English. As English is not among the official languages of Belgium (French, Dutch, German), but it had to be the language of the study since the interviewer was not fluent in these languages, it is possible that some specialists did not participate for linguistic reasons. In fact, two eligible specialists were excluded because they could not be interviewed in English. This might also be the reason for the under-representation of the French speaking community of Belgium (Wallonia). Consequently, whether or not the results apply to non-English speaking professionals is unknown. A third limitation might be a sample bias towards male participants with age 30–49 years. The age and gender demographics of the total population remains unknown, so their effect remains unassessed.

Another limitation concerns our interview methodology. Rather than allowing participants to freely elaborate on their general perceptions on IPC, we have evolved the conversations around 10 principal thematic questions. Therefore, it is possible that IPC perceptions not directly linked to our themes of interest have not been documented.

The last limitation concerns the generalizability of the results. This study has been confined to CHF/COPD specialists in inpatient setting. It is not clear whether the documented perception would extend to other types of chronic disease, general practitioners, PC experts and nurses and to health-care personnel working in outpatient settings. Additionally, even within the European Union (EU), healthcare systems and practices vary considerably. Consequently, one should be careful before generalizing our hospital based findings to different countries, even within the EU or to other care settings.

## Conclusions

Aspects of IPC appear to be embodied alongside curative treatment, however, PC experts are not usually involved and therefore current IPC practices are probably not optimal. Misconceptions about PC and its association to death/end-of-life seem to be significant reasons for the limited participation of PC specialists and the late initiation of PC itself.

Healthcare organizations in Europe and worldwide acknowledge the benefits that patients with CHF/COPD can receive from IPC. Targeted education and improved communication could increase the awareness of all involved parties on aspects of IPC and thus lead to improved practices and better quality of life for the patients.

## Abbreviations

ACP: Advance care planning
CHF: Chronic Heart Failure
COPD: Chronic Obstructive Pulmonary Disease
EU: European Union
IPC: Integrated palliative care
MDT: Multidisciplinary team
PC: Palliative care
RCT: Randomized Controlled Trials

## Footnotes

1 Throughout the manuscript, integration of PC refers to the process of PC evolving into integrated. On the other hand, provision of PC concerns the administration of PC services to the patient.
